# Characterization of Global Transcriptome Using Illumina Paired-End Sequencing and Development of EST-SSR Markers in Two Species of *Gynostemma* (*Cucurbitaceae*)

**DOI:** 10.3390/molecules201219758

**Published:** 2015-11-30

**Authors:** Yue-Mei Zhao, Tao Zhou, Zhong-Hu Li, Gui-Fang Zhao

**Affiliations:** 1 Key Laboratory of Resource Biology and Biotechnology in Western China (Ministry of Education), College of Life Sciences, Northwest University, 229 Taibai Bei Road, Xi’an 710069, China; yezi19820320@163.com (Y.-M.Z.); woody196@163.com (T.Z.); lizhonghu@nwu.edu.cn (Z.-H.L.); 2 College of Biopharmaceutical and Food Engineering, Shangluo University, Beixin Street, Shangluo 726000, China

**Keywords:** *Gynostemma*, transcriptome, illumina paired-end sequencing, *de novo* assembly, EST-SSR

## Abstract

*Gynostemma pentaphyllum* is an important medicinal herb of the *Cucurbitaceae* family, but limited genomic data have hindered genetic studies. In this study, transcriptomes of two closely-related *Gynostemma* species, *Gynostemma cardiospermum* and *G. pentaphyllum*, were sequenced using Illumina paired-end sequencing technology. A total of 71,607 nonredundant unigenes were assembled. Of these unigenes, 60.45% (43,288) were annotated based on sequence similarity search with known proteins. A total of 11,059 unigenes were identified in the Kyoto Encyclopedia of Genes and Genomes Pathway (KEGG) database. A total of 3891 simple sequence repeats (SSRs) were detected in 3526 nonredundant unigenes, 2596 primer pairs were designed and 360 of them were randomly selected for validation. Of these, 268 primer pairs yielded clear products among six *G. pentaphyllum* samples. Thirty polymorphic SSR markers were used to test polymorphism and transferability in *Gynostemma*. Finally, 15 SSR makers that amplified in all 12 *Gynostemma* species were used to assess genetic diversity. Our results generated a comprehensive sequence resource for *Gynostemma* research.

## 1. Introduction

*Gynostemma* (*Cucurbitaceae*), is a genus of perennial creeping herbs with both sexual reproduction and clonal growth by rhizomes or bulbils [1]. This genus has around 16 species and two varieties, distributed in forests, scrubs and bush habitats at 60–3200 m elevations throughout China, India, Myanmar, Korea and Japan [2]. Drainage areas of the Yangtze River in Southwest China’s Yunnan Province are thought to be the modern distribution center of this genus. *Gynostemma* can be divided into two subgenera (*Gynostemma* and *Triostellum*) according to different fruit morphology [2]. In recent years, *Gynostemma* has been attracting attention since they contain saponins, amino acids, and reducing sugars, which could be commercially useful. For example, *Gynostemma pentaphyllum*, widespread as a traditional Chinese medicinal herb, is thought to inhibit tumor cell growth, anti-ulceration and to enhance immunity [3,4]. Approximately 84 dammarane-type saponin glycosides were found in *G. pentaphyllum* [5], some of them have structural similarities to glycosides found in *Panax ginseng* C.A. Mey [6], but different saponin contents were observed in various other species of *Gynostemma* [7]. Natural populations of *Gynostemma* were destroyed in recent years due to excessive harvesting, especially *G. pentaphyllum,* which has been listed as a Grade II Key Protected Wild Plant Species by the Chinese Government [8]. It is necessary to preserve natural stocks of *Gynostemma* spp., and assess their genetic diversity and differentiation. Molecular genetic research of *Gynostemma* is limited [9] because most studies have mainly focused on extracting bioactive components [5,10,11,12,13]. Subramaniyam *et al.* (2011) [14] reported *de novo* transcriptome assembly of *G. pentaphyllum* with Roche platforms using the materials of leaves and roots, but the research focused on the identification of secondary metabolite genes. So far, only 14 genomic simple sequence repeats (SSRs) and 14 inter-simple sequence repeats (ISSRs) have been exploited in *Gynostemma* [15,16]. Thus, more markers are needed to better understand the genetic diversity and to develop conservation strategies for *Gynostemma*.

SSR markers have turned out to be an effective tool for germplasm characterization and genetic diversity studies. SSRs can be divided into two categories based on the original sequences used for development of SSRs: genomic SSRs and expressed sequence tag (EST)-SSRs. Developing genomic SSR markers from random genomic sequences is labor, money and time intensive [17,18]. On the contrary, EST-SSRs identified from transcribed RNA sequences are more conserved than noncoding sequences. EST-SSRs are becoming more and more widespread, not only because they are potentially linked with particular transcriptional regions that contribute to agronomic phenotypes [19,20], but also because they have high transferability among closely-related species [21,22,23,24,25,26]. With the development of next-generation sequencing (NGS), it has become possible to generate large numbers of transcriptomic datasets for nonmodel organisms [27] using various platforms such as Roche 454, Illumina HiSeq, and Applied Biosystems SOLiD. Obtaining large numbers of valuable EST sequences via NGS is important for gene annotation and discovery [28,29], comparative genomics [30], development of molecular markers [31,32], and population genomics studies of genetic variation linked to adaptive traits [33]. Recently, an increasing number of EST datasets have become available for model and non-model organisms, but only a limited number of *Gynostemma* EST sequences are available in the public database.

In this study, we describe the generation, *de novo* assembly, and annotation of a transcriptome-derived EST dataset using Illumina paired-end sequencing technology from two *Gynostemma* species, *G. pentaphyllum* and *G. cardiospermum*. In addition, we mined and validated a large set of EST-SSR markers and investigated the genetic relationship among 12 selected species. This EST datasets will serve as a valuable genomic resource for further studies in *Gynostemma*, e.g., novel gene discovery and marker-assisted selective breeding.

## 2. Results

### 2.1. Assembly of Gynostemma Transcriptome Data from Illumina Sequencing

**Figure 1:**
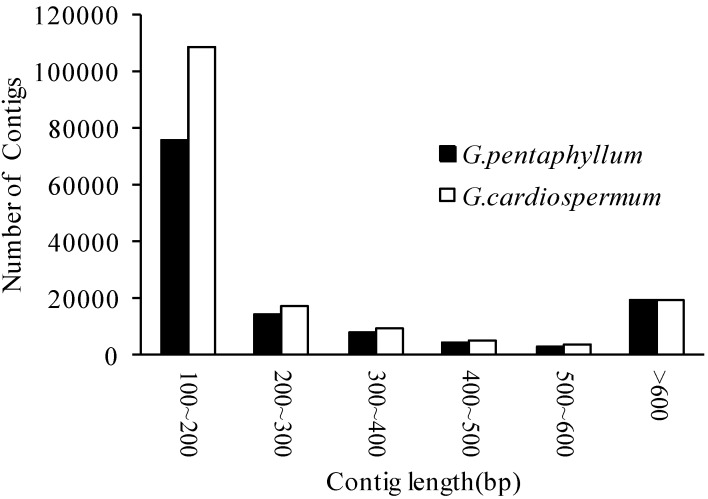
Frequency distribution of the contig sizes from two *Gynostemma* species. The frequency distribution of contig sizes resulting from Illumina HiSeq™ 2000 sequencing, as assembled using Trinity.

After stringent quality assessment and data filtering, Illumina HiSeq™ 2000 sequencing generated 43,175,448 high-quality reads for *Gynostemma pentaphyllum* and 52,782,146 high-quality reads for *Gynostemma cardiospermum*, respectively. The raw data were deposited in the NCBI Sequence Read Archive (SRA) under the accession number SRA305674. Using the Trinity assembler software [34], short-read sequences from *G. pentaphyllum* and *G. cardiospermum* were assembled *de novo* into 1,488,035 contigs and 1,911,378 contigs, respectively. Transcriptome reads and assembled contigs information for two *Gynostemma* species are shown in Table 1. The frequency distribution of contigs length from these two *Gynostemma* species showed little difference, except for 100–200 bp, which showed more contigs in *G. cardiospermum* (Figure 1). Using paired end-joining, gap-filling, and Trinity, these contigs were assembled into scaffolds which were further assembled into unigenes. Finally, we obtained 40,257 and 44,000 unigenes from *G. pentaphyllum* and *G. cardiospermum*, respectively (Table 2). In addition, we obtained a nonredundant set of unigene sequences by pooling contigs from the two species and assembling them together into 71,607 unigenes. As shown in Table 2, the 71,607 nonredundant unigenes were used for *in silico* mining and validation of genic-SSR markers. Among the combined 71,607 nonredundant sequences, there were 51,250 (71.57%) that ranged from 200 to 1000 bp, 12,852 (17.95%) from 1000 to 2000 bp, and 7505 (10.48%) greater than 2000 bp.

**Table 1:** Transcriptome reads and assembled contigs information for two *Gynostemma* species.

Species	Total Reads	Total Clean Nucleotides (Nt)	Q30 Percentage	GC Percentage		Total Number of Contigs	Total Length of Contigs (Nt)	N50 of Contigs	Mean (Nt)
*G. pentaphyllum*	43,175,448	4,360,277,191	80.16%		43.55%	1,488,035	110,745,998	71	74
*G. cardiospermum*	52,782,146	5,330,256,148	82.69%		44.00%	1,911,378	136,726,651	66	71

**Table 2:** Summary of the unigenes from two *Gynostemma* species.

Unigene Source	Total Number of Unigenes	Total Length of Unigenes (Nt)	Mean Length of Unigenes (Nt)	N50 of Unigenes
*G. pentaphyllum*	40,257	35,161,843	873.43	1516
*G. cardiospermum*	44,000	37,234,004	846.23	1504
All	71,607	61,367,129	857.00	1535

### 2.2. Functional Annotation and Classification

**Figure 2:**
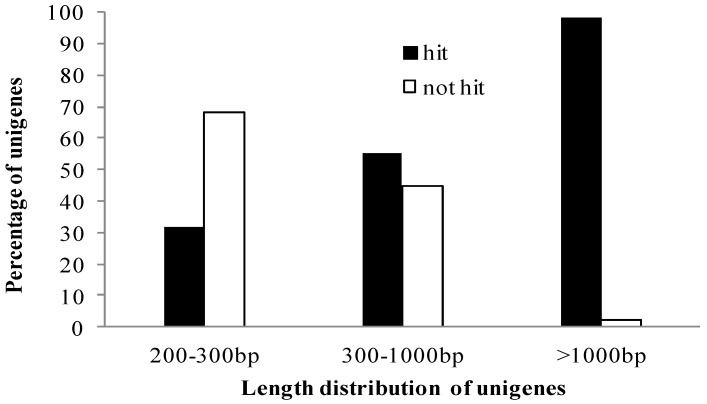
Comparison of unigene length between hit and not-hit unigenes. Longer unigenes were more likely to have BLAST matches in protein databases.

**Figure 3:**
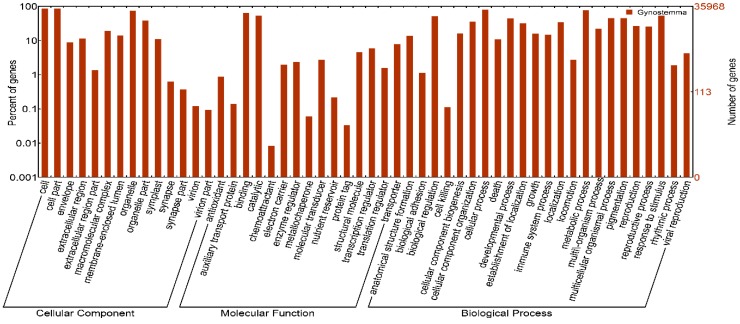
Gene Ontology classification of assembled unigenes. The results are summarized in three main categories: Biological Process, Cellular Component and Molecular Function. In total, 35,968 unigenes with BLAST matches to known proteins were assigned to gene ontology terms.

**Figure 4:**
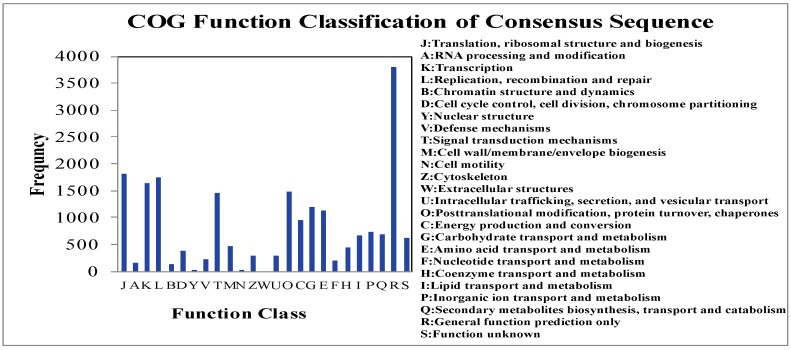
Histogram presentation of clusters of orthologous groups (COG). In total, 20,585 sequences were assigned to 25 COG classifications.

**Figure 5:**
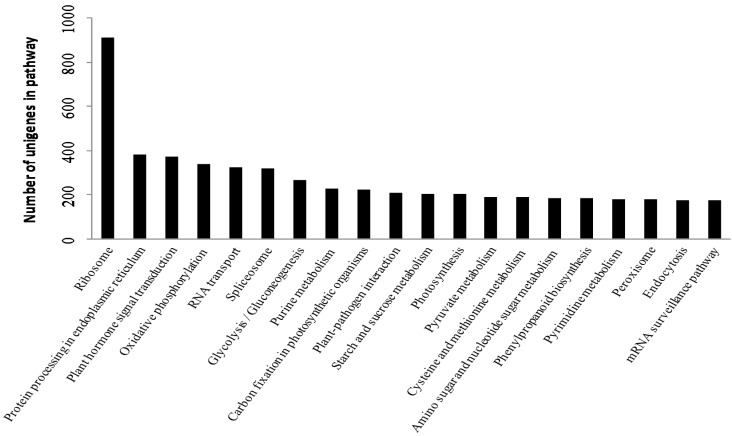
Kyoto Encyclopedia of Genes and Genomes (KEGG) classification of unigenes. 5,443 unigenes were assigned to the top 20 pathways in KEGG.

A homology-based approach was conducted for validation and annotation of assembled unigenes. Among 71,607 unigenes, 60.28% (43,167) showed homology in the nonredundant (nr) database, and 46.04% (32,970) unigenes had BLAST hits in Swiss-Prot database. A total of 60.45% (43,288) unigenes were successfully annotated in the nr and/or Swiss-Prot databases. Additionally, 97.73% (19,895 of 20,357) of the unigenes over 1000 bp in length showed homologous matches, whereas only 32.33% (6619 of 20,474) of the unigenes shorter than 300 bp showed matches (Figure 2). The unigenes homologous to known protein sequences in nr database were further assigned to gene ontology (GO) terms using Blast2GO. A total of 35,968 unigenes were assigned to 549,570 GO term annotations, which belonged to biological processes, molecular functions and cellular components clusters (Figure 3). Among biological processes, “cellular process” was the most dominant group, followed by “metabolic process”, “response to stimulus”, and “biological regulation”. Regarding the molecular functions category, the major GO terms were “binding” and “catalytic activity”. Under the cellular components category, “cell part” and “cell” represented the most abundant classification, followed by “organelle” and “organelle part”. All unigenes were searched against the COG database to predict possible functions and phylogenetically classify orthologous gene products. Out of 43,167 nr hits, 20,585 sequences were assigned to one or more COG classifications (Figure 4). Among the 25 COG categories, the cluster for “general function prediction” was the largest group, followed by “translation ribosomal structure and biogenesis”, “replication, recombination and repair”, and “posttranslational modification, protein turnover, chaperones”. In contrast, only a few unigenes were assigned to “nuclear structure and extracellular structure”. According to the Kyoto Encyclopedia of Genes and Genomes (KEGG) database, 11,059 unigenes were identified with pathway annotation and were assigned to 117 KEGG pathways (Table S1). The top 20 pathways, including 5443 unigenes, are listed in Figure 5. The most highly represented pathways were “Ribosome”, followed by “Protein processing in endoplasmic reticulum” and “Plant hormone signal transduction”. Being important medicinal plants used in China, previous research on *Gynostemma* spp. has mostly focused on saponins biosynthesis pathways. As expected, some key genes encoding enzymes related to the synthesis of triterpene compounds, which are important components of saponins, were revealed in metabolism pathway. For example, the genes involved in mevalonate (MVA) and 2-*C*-methyl-d-erythritol 4-phosphate (MEP) pathways were identified in our study (Table 3) and these genes may provide valuable resources for research on gynosaponin biosynthesis.

**Table 3:** List of triterpene saponin biosynthesis-related genes in the *Gynostemma* transcriptome.

Gene ID	Length	KO ID	Annotation
T3_Unigene_BMK.23540	2373	K01662	1-deoxyxylulose-5-phosphate synthase, (EC:2.2.1.7)
T3_Unigene_BMK.25990	2720	K01662	1-deoxyxylulose-5-phosphate synthase, (EC:2.2.1.7)
T3_Unigene_BMK.37882	301	K01662	1-deoxyxylulose-5-phosphate synthase, (EC:2.2.1.7)
T4_Unigene_BMK.23719	2489	K01662	1-deoxyxylulose-5-phosphate synthase, (EC:2.2.1.7)
T4_Unigene_BMK.30793	3057	K01662	1-deoxyxylulose-5-phosphate synthase, (EC:2.2.1.7)
T4_Unigene_BMK.33182	2483	K01662	1-deoxyxylulose-5-phosphate synthase, (EC:2.2.1.7)
CL10430Contig1	2754	K01662	1-deoxyxylulose-5-phosphate synthase, (EC:2.2.1.7)
T3_Unigene_BMK.15665	2671	K01662	1-deoxyxylulose-5-phosphate synthase, (EC:2.2.1.7)
CL9143Contig1	1586	K00919	4-diphosphocytidyl-2-*C*-methyl-d-erythritol kinase, (EC:2.7.1.148)
T3_Unigene_BMK.22099	359	K03526	4-hydroxy-3-methylbut-2-en-1-yl diphosphate synthase, (EC:1.17.7.1)
T3_Unigene_BMK.28355	906	K03527	4-hydroxy-3-methylbut-2-enyl diphosphate reductase, (EC:1.17.1.2)
CL12440Contig1	616	K03527	1-hydroxy-2-methyl-2-(E)-butenyl 4-diphosphate reductase, (EC:1.17.1.2)
T3_Unigene_BMK.9388	411	K03527	1-hydroxy-2-methyl-2-(E)-butenyl 4-diphosphate reductase, (EC:1.17.1.2)
CL12699Contig1	1973	K01823	isopentenyl-diphosphate delta-isomerase, (EC:5.3.3.2)
T4_Unigene_BMK.25472	575	K01823	isopentenyl-diphosphate delta-isomerase, (EC:5.3.3.2)
CL7433Contig1	750	K01823	isopentenyl-diphosphate delta-isomerase, (EC:5.3.3.2)
T3_Unigene_BMK.21000	1177	K13789	geranyl geranyl pyrophosphate synthase, (EC:2.5.1.29)
T4_Unigene_BMK.20525	1569	K14066	geranylgeranyl pyrophosphate synthase, (EC:2.5.1.30)
T3_Unigene_BMK.18996	1754	K14066	geranylgeranyl pyrophosphate synthase, (EC:2.5.1.30)
T3_Unigene_BMK.35842	305	K00626	acetyl-CoA acetyltransferase, mitochondrial, (EC:2.3.1.9)
T3_Unigene_BMK.35857	278	K00626	acetyl-CoA acetyltransferase, mitochondrial, (EC:2.3.1.9)
T4_Unigene_BMK.23349	1785	K00626	acetyl-CoA C-acetyltransferase, (EC:2.3.1.9)
T3_Unigene_BMK.18569	1657	K00626	acetyl-CoA C-acetyltransferase, (EC:2.3.1.9)
CL11048Contig1	2014	K01641	hydroxymethylglutaryl-CoA synthase, (EC:2.3.3.10)
T3_Unigene_BMK.2712	475	K00021	hmg-CoA reductase, (EC:1.1.1.34)
CL14352Contig1	2368	K00021	hmg-CoA reductase, (EC:1.1.1.34)
T4_Unigene_BMK.28606	1468	K00021	hmg-CoA reductase, (EC:1.1.1.34)
T4_Unigene_BMK.28692	322	K00021	hmg-CoA reductase, (EC:1.1.1.34)
T3_Unigene_BMK.22340	296	K00021	hmg-CoA reductase, (EC:1.1.1.34)
T3_Unigene_BMK.3271	467	K00869	mevalonate kinase, (EC:2.7.1.36)
CL13115Contig1	526	K00869	mevalonate kinase, (EC:2.7.1.36)
T4_Unigene_BMK.23148	1576	K00869	mevalonate kinase, (EC:2.7.1.36)
T4_Unigene_BMK.26974	522	K00869	mevalonate kinase, (EC:2.7.1.36)
T3_Unigene_BMK.14474	1691	K00869	mevalonate kinase, (EC:2.7.1.36)
T3_Unigene_BMK.22024	1903	K00938	Phosphomevalonate kinase, (EC:2.7.4.2)
T4_Unigene_BMK.29327	2282	K01597	diphosphomevalonate decarboxylase, (EC:4.1.1.33)
T3_Unigene_BMK.15864	1831	K01597	diphosphomevalonate decarboxylase, (EC:4.1.1.33)
T3_Unigene_BMK.5322	215	K11778	undecaprenyl pyrophosphate synthetase, (EC:2.5.1.31)
T4_Unigene_BMK.33183	1237	K11778	undecaprenyl pyrophosphate synthetase, (EC:2.5.1.31)
T4_Unigene_BMK.21428	1774	K00801	farnesyl-diphosphate farnesyltransferase, (EC:2.5.1.21)
T3_Unigene_BMK.3061	271	K00511	Squalene monooxygenase, (EC:1.14.99.7)
CL1336Contig1	2048	K00511	Squalene monooxygenase, (EC:1.14.99.7)

KO: KEGG Orthology.

### 2.3. Frequency and Distribution of Different Types of SSR Markers

**Figure 6:**
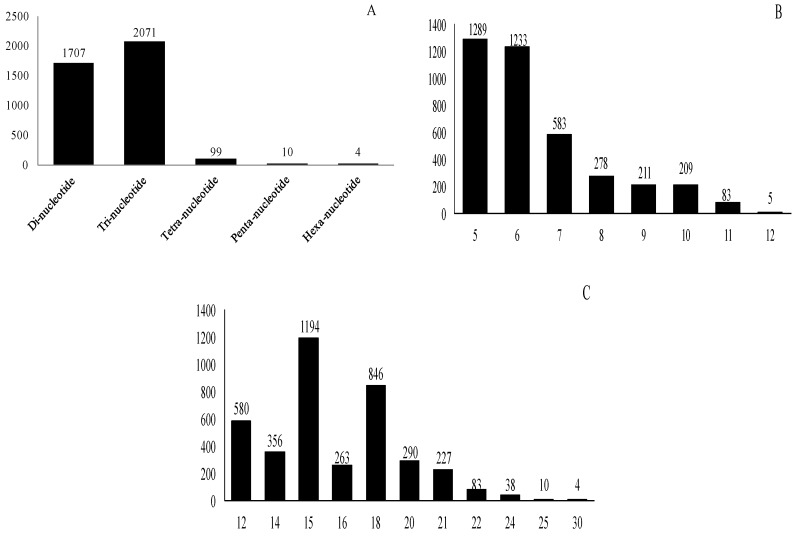
Frequency distribution of the *Gynostemma* expressed sequence tag (EST)-SSRs of different sizes. (**A**) Unit size; (**B**) Number of repeats; (**C**) SSR locus length.

A total of 3891 SSR loci were identified from 3526 nonredundant unigenes, representing 4.92% of the total 71,607 unigenes. The distribution density was one SSR locus per 15.78 kb. This study excluded mononucleotide repeats and complex SSRs. There were 329 unigenes with more than one SSR locus. The most frequent repeat unit in nonredundant unigenes were trinucleotides followed by dinucleotides (Figure 6A); and di- and tri-nucleotide repeats constituted 3778 (97.10%) of the identified SSR loci. The number of reiterations of a given repeat unit ranged from five to 12 and SSRs with five reiterations were the most abundant (Figure 6B). The majority of the SSR sequences were from 12 to 21 bp in length, accounting for 96.53% (3756) of the total identified SSR loci; SSR loci with a length of 15 bp were the most frequent. The longest SSR locus was 30 bp (Figure 6C). More details about different repeat motif of di- and trinucleotide repeats in EST-SSRs are listed in Table 4. The dominant di- and tri-nucleotide repeat motif in SSRs were AG/CT and AAG/CTT respectively. There was only one CG/CG repeat motif and very few ACT/AGT repeats in our results (Table 4).

**Table 4:** Frequency distribution of the di- and tri-nucleotide repeat motifs in the *Gynostemma*.

Serial No.	Repeat Motif	Number of Reiterations of the Motif								Total
		5	6	7	8	9	10	11	12	
1	AG/CT	#	456	285	217	189	192	81	3	1423
2	AAG/CTT	576	358	137	6	0	0	0	0	1077
3	ATC/ATG	159	74	25	0	0	0	0	0	258
4	AT/AT	#	95	54	36	18	14	2	1	220
5	AAT/ATT	97	50	19	2	0	0	0	0	168
6	AGG/CCT	90	41	12	1	0	0	0	0	144
7	AGC/CTG	83	24	9	1	0	0	0	0	117
8	ACC/GGT	52	32	6	1	0	0	0	0	91
9	AAC/GTT	49	24	14	2	0	0	0	0	89
13	CCG/CGG	49	17	3	0	0	0	0	0	69
10	AC/GT	#	28	17	10	4	3	0	1	63
11	ACG/CGT	24	10	0	0	0	0	0	0	34
12	ACT/AGT	15	5	2	2	0	0	0	0	24
14	CG/CG	0	1	0	0	0	0	0	0	1
15	Other motifs *	95	18	0	0	0	0	0	0	113
	Total	1289	1233	583	278	211	209	83	5	3891

Note: * indicated tetra-, penta-, and hexa-nucleotide motifs in our study; # means that this item was not considered when detecting EST-SSRs.

### 2.4. Development, Validation and Transferability of SSR Markers

To further evaluate the assembly quality, 2586 primer pairs (Table S2) were designed using Primer 3.0 based on 3891 SSR loci generated from MISA. Primer pairs for the remaining 1305 SSR loci could not be designed successfully because their flanking sequences were either too short or the nature of sequences did not satisfy the criteria of BatchPrimer3 v 1.0 software. All 2586 unigene sequences were subjected to BLAST analysis to predict the likely function of these EST-SSRs. There were 2559 transcriptome sequences that showed homology to functional loci of other plants (Table S2). From the 2586 primer pairs, 360 (Table S3) were randomly selected for validation using DNA from the six samples of *G. pentaphyllum* of three different populations. Among the 360 primer pairs, 268 were successfully amplified via PCR. The remaining 92 primer pairs failed to generate PCR products, even when the annealing temperature was reduced by 8 °C. Of the 268 working primer pairs, 239 amplified products of the expected size including 23 monomorphic loci and 216 polymorphic loci among six genotypes of *G. pentaphyllum*. The other 29 generated larger products than the expected size, suggesting that there may exist introns in the amplifying regions. To test interspecies transferability across 12 related species (26 individuals) in the genus *Gynostemma*, 30 SSR pairs were selected from the 216 microsatellites that produce polymorphic size fragments. Of the 30 polymorphic SSRs, 15 primer pairs could amplify PCR products and show polymorphic fragments from all 12 *Gynostemma* species (Table 5 and Table 6). Four pairs of primers (G-EST-SSR93, G-EST-SSR29, G-EST-SSR55, and G-EST-SSR31) failed to produce PCR fragments in *Gynostemma laxiflorum*; five pairs (G-EST-SSR85, G-EST-SSR42, G-EST-SSR54, G-EST-SSR59, and G-EST-SSR51) failed to produce PCR fragments in *Gynostemma caulopterum*; one pair of primers (G-EST-SSR14) failed to produce PCR fragments in *Gynostemma laxum* and *G. caulopterum*; one pair of primers (G-EST-SSR7) failed to produce PCR fragments in *Gynostemma pubescens*, *G. laxum*, *G. caulopterum*, and *G. laxiflorum*; one pair of primers (G-EST-SSR62) failed to produce PCR fragments in *Gynostemma microspermum*, *G. pubescens*, *G. laxum*, *G. laxiflorum*, and *G. caulopterum*; and three pairs of primers (G-EST-SSR158, G-EST-SSR92, and G-EST-SSR3) failed to produce PCR fragments in *G. laxum*. The cross-species amplification of these 30 EST-SSRs developed from *G. pentaphyllum* and *G. cardiospermum* in 10 additional *Gynostemma* species (*G. pentaphyllum* and *G. cardiospermum* were not considered when calculating the transferability of the EST-SSRs) was 92.33% in 300 combinations tested (30 SSRs × 10 species).

**Table 5:** Details of 15 genic-SSR loci showing polymorphism among 12 *Gynostemma* species.

Primer Name	Base on Sequence ID	SSRs	EPS (bp)	OPS (bp)	Alleles	PIC
G-EST-SSR19	CL10682Contig1	(AT)6	149	149–161	5	0.67
G-EST-SSR20	CL10765Contig1	(GAA)5	151	148–163	8	0.82
G-EST-SSR40	CL11560Contig1	(TCT)5	151	151–160	4	0.68
G-EST-SSR44	CL13255Contig1	(AG)6	164	162–196	11	0.87
G-EST-SSR47	CL13343Contig1	(TCT)6	148	151–172	8	0.79
G-EST-SSR57	CL14153Contig1	(TGA)5	149	152–170	6	0.74
G-EST-SSR75	CL400Contig1	(TCA)6	172	172–202	7	0.80
G-EST-SSR76	CL435Contig1	(TCT)5	131	122–137	6	0.76
G-EST-SSR89	CL1983Contig1	(GAAA)5	146	146–166	6	0.79
G-EST-SSR100	CL2782Contig1	(GAA)6	145	136–163	9	0.79
G-EST-SSR118	CL8580Contig1	(CGG)5	141	138–153	6	0.62
G-EST-SSR131	CL9957Contig1	(TCT)5	149	149–158	5	0.55
G-EST-SSR140	CL10320Contig1	(CTA)6	158	158–170	6	0.73
G-EST-SSR306	CL11525Contig1	(CT)7	169	169–189	6	0.73
G-EST-SSR316	CL12699Contig1	(GAA)5	135	135–159	8	0.66
	Mean	-	-	-	6.73	0.73

EPS: expected product size; OPS: observed product size.

**Table 6:** The 26 individual plants (belonging to 12 species) used for validation and the genetic diversity study.

No.	Species	Locality, Province	Latitude (N), longitude (E)	Characteristics
1	*G. pentaphyllum*	Ankang, Shaanxi	32°25′N,109°04′E	wild
2	*G. pentaphyllum*	Ankang, Shaanxi	32°25′N,109°04′E	Cultivar
3	*G. pentaphyllum*	Kunming, Yunnan	25°14′N,102°49′E	Wild
4	*G. pentaphyllum*	Kunming, Yunnan	25°14′N,102°49′E	Cultivar
5	*G. pentaphyllum*	Panzhihua, Sichuan	26°36′N,101°43′E	Wild
6	*G. pentaphyllum*	Panzhihua, Sichuan	26°36′N,101°43′E	Cultivar
7	*G. burmanicum var. molle*	Dehong, Yunnan	24°48′N, 98°17′E	Wild
8	*G. burmanicum*	Menghai, Yunnan	22°02′N, 100°22′E	Wild
9	*G. burmanicum*	Dehong, Yunnan	24°36′N, 97°39′E	Wild
10	*G.pentaphyllum var. dasycarpum*	Mengla, Yunnan	22°14′N, 101°15′E	Wild
11	*G. pentaphyllum var. dasycarpum*	Jinghong, Yunnan,	22°01′N, 100°45′E	Wild
12	*G. longipes*	Xuancheng, Anhui	30°55′N,118°46′E	Wild
13	*G. longipes*	Ankang, Shaanxi	32°25′N,109°22′E	Wild
14	*G. longipes*	Enshi, Hubei	30°03′N, 109°49′E	Wild
15	*G. longipes*	Zhaotong, Yunnan	27°43′N,103°54′E	Wild
16	*G. pubescens*	Menghai, Yunnan	21°56′N, 100°36′E	Wild
17	*G. pubescens*	Menglun, Yunnan	21°56′N, 101°14′E	Wild
18	*G. pubescens*	Yingjiang, Yunnan	24°42′N, 97°55′E	Wild
19	*G. pubescens*	Enshi, Hubei	30°18′N, 109°31′E	Wild
20	*G. laxum*	Jiujiang, Jiangxi,	29°17′N, 115°07′E	Wild
21	*G. microspermum*	Pu’er, Yunnan	23°07′N, 100°22′E	Wild
22	*G. laxiflorum*	Xuancheng, Anhui	30°41′N,118°39′E	Wild
23	*G. yixingense*	Tongling, Anhui	30°57′N,117°48′E	Wild
24	*G. caulopterum*	Renhuai, Guizhou	27°48′N, 106°26′E	Wild
25	*G. cardiospermum*	Ankang, Shaanxi	32°13′N,109°01′E	Wild
26	*G. cardiospermum*	Ankang, Shaanxi	32°13′N,109°01′E	Wild

### 2.5. Genetic Diversity and Relatedness in the Genus Gynostemma

**Figure 7:**
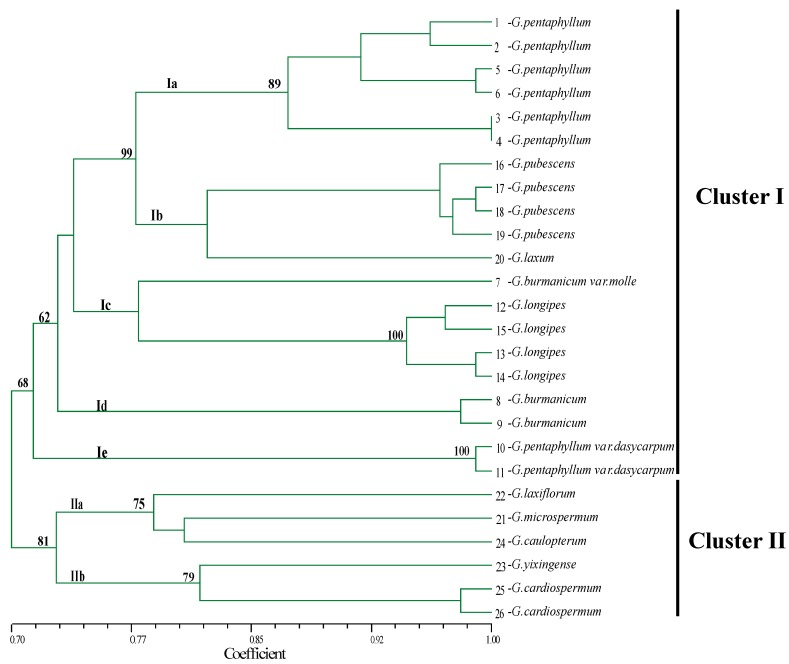
Genetic relationships among *Gynostemma* species based on EST-SSR markers. Genetic relationships among 26 individual plants. The scale at the bottom of the dendrogram indicates the level of similarity between the genotypes. Bootstrap values (>50) were labeled on the branches from 1000 re-samplings.

The 15 primer pairs that yielded clear, highly polymorphic bands from all *Gynostemma* species were used to assess the genetic diversity in a set of 26 individual plants representing 12 species of *Gynostemma* (Table 5 and Table 6). A total of 101 alleles were identified, the number of alleles per locus ranged from four to 11 with an average of 6.73 alleles. Polymorphism information content (PIC) ranged from 0.55 to 0.87 with an average of 0.73, suggesting that the developed EST-SSRs were highly polymorphic. A phenogram based on Jaccard’s similarity coefficients was constructed to resolve the relationship of 26 individuals from 12 species (Figure 7), which showed two distinct clusters at a cut-off similarity index of 0.71. Cluster I contained seven species, which corresponded to *subgen. Gynostemma*, and was divided into five sub-clusters: Ia, Ib, Ic, Id and Ie, at a cut-off similarity index of 0.78; Sub-cluster Ia comprised six *G. pentaphyllum* genotypes (three wild and three cultivar genotypes); Sub-cluster Ib comprised four *G. pubescens* from four populations and one *G. laxum*; Sub-cluster Ic comprised four *G. longipes* from four populations and one *G. burmanicum var. molle*; Sub-cluster Id comprised two *G. burmanicum* from two populations; and Sub-cluster Ie comprised two *G. pentaphyllum* *var. dasycarpum* from Jinghong and Mengla locations, respectively. Cluster II included five species that corresponded to *subgen. Triostellum*, and was divided into two sub-clusters, IIa and IIb, at a cut-off similarity index of 0.79. Sub-cluster IIa comprised one *G. microspermum*, one *G. laxiflorum* and one *G. caulopterum*, and Sub-cluster IIb comprised one *G. yixingense* and two *G. cardiospermum* individuals.

## 3. Discussion

### 3.1. Functional Annotation of Unigenes

Presently, most research concentrate on isolating bioactive components from *Gynostemma* spp., but the potential molecular mechanisms producing such compounds is still unclear. Transcriptome sequencing is an effective method for novel gene discovery and SSR marker development. In this study, 71,607 nonredundant unigenes were obtained after assembly. In total, 60.45% (43,288) of all unigenes had homologs in the NCBI nr or Swiss-Prot protein databases, which was lower than that reported by Subramaniyam *et al.* [14] with leaves and roots as sequencing materials in *Gynostemma* *pentaphyllum*. Compared with other plants used in Chinese medicine, this was higher than *Epimedium* *sagittatum* [35], but lower than *Panax quinquefolius* [36] and *Panax notoginseng* [37]. Among the 43,288 unigenes with BLAST matches in the NCBI nr or Swiss-Prot protein database, 97.73% were over 1000 bp, whereas unigenes shorter than 300 bp in length only accounted for 32.33% of the total. Therefore, we infer that the large proportion of BLAST matches in *Gynostemma* was probably due to the large number of long sequences in our unigene database, which was also validated in other plants [38,39,40]. Perhaps the lack of a characterized protein domain, a common feature of the shorter unigene sequences, was the cause of the small number of shorter sequences showing BLAST hits in the protein databases. Further research with GO analysis revealed that most genes are involved in many biological processes in *Gynostemma*. Many genes were assigned to “metabolic process” and “catalytic activity” classes, which suggest a great deal of enzymes involved in primary and secondary metabolism. Among the KEGG pathways, the well-represented pathways discovered in our study were “Ribosome”, “Protein processing in endoplasmic reticulum” and “Plant hormone signal transduction”. Furthermore, some key genes involved in the biosynthesis of terpenoids were identified, several of which were found in other species [36,37]. Compared with the transcriptome of leaves and roots, genes related to biosynthesis of terpenoids showed little difference. For instance, 4-hydroxy-3-methylbut-2-en-1-yl diphosphate synthase (EC: 1.17.7.1), farnesyl-diphosphate farnesyltransferase (EC: 2.5.1.21) and Squalene monooxygenase (EC: 1.14.99.7), which were found in our study, were not presented in the results of Subramaniyam *et al.* [14]. Likewise, the genes present in results of Subramaniyam *et al.* [14], Hydroxymethylglutaryl-CoA reductase (EC: 1.1.1.88), Geranyl transtransferase (EC: 2.5.1.10), Dimethylallyltranstransferase (EC: 2.5.1.1), *etc.*, did not appear in our study either. These results reflect that there might exist different transcriptomic signatures in different tissues. Some unigenes without BLASTx hits may be potential *Gynostemma*-specific genes. Both of these classes can provide valuable information for the further study of *Gynostemma* spp., such as novel gene discovery and cloning, functional studies, and metabolic engineering of enzymes.

### 3.2. SSR Marker Frequency and Distribution in Gynostemma Transcriptome

Polymorphic SSRs play an important role in genetic diversity research, genetic mapping studies, comparative genomics, and marker-assisted selection breeding [41]. Transcriptomics provides a rich source for SSR discovery because it generates plenty of sequences. A total of 3891 SSRs greater than 12 bp in length were identified from 3526 nonredundant unigenes, 4.92% of the total 71,607 unigenes possessed SSRs. It is obvious that the SSR frequency detected in *Gynostemma* is in accordance with the range of frequencies (2.65%–16.82%) reported before for other dicotyledonous species [42]. Several factors affect the EST-SSR frequency. First, the criteria for calling microsatellites is the most important factor of EST-SSR frequency, e.g., the repeat length threshold and the number of repeat motifs. Most studies have excluded the mononucleotide repeat motifs because they may result from sequencing errors. Some studies take three-repeat units into account when calculating the number of dinucleotide repeat units [40], while others do not [43,44,45]. In addition, we identified SSRs primarily from unigenes over 1000 bp, which may reduce the frequency to a certain degree. Secondly, genome structure or composition could also influence SSR frequency [46]. For example, it is reported that the small genome size of rice was the cause of the high frequency of EST-SSR sequences [47]. Finally, the different software used to detect SSR loci can also affect the SSR frequency. 

Theoretically, the frequency of di-, tri-, tetra-, penta-, and hexanucleotide repeats should be in turn decreased according to the relative probability of replication slippage events [48]. The most abundant type of repeat motif among the *Gynostemma* unigenes analyzed was trinucleotide (Figure 6A). This finding is consistent with the earlier results reported before [22,35,45,48,49,50,51,52,53,54], which showed the trinucleotide motif is the most frequent repeat type. Some studies point out the reason for the high frequency of tri-nucleotide SSRs is that the selection against frameshift mutations might limit the expansion of other SSR types [43,55,56,57]. Meanwhile, other studies show that the most abundant class of SSRs was dinucleotide [38,39,42,58,59]. There are also some plant species showing approximately equal proportions of dinucleotide and tri-nucleotide repeats in their transcriptome sequences, e.g., *Aspidistra saxicola* [44], sweet potato [39], and oak [60]. The most frequent repeats of di- and tri-nucleotide were AG/CT and AAG/CTT, respectively, which was in accordance with the reports in sesame [38], oil palm [61], sweet potato [39], *Primula* [62], and *Nothofagus nervosa* [63]. 

### 3.3. Transferability of SSR Markers and Genetic Relationships among Different Species of Gynostemma

In this study, 3891 SSR markers were developed and 360 primer pairs were randomly selected to evaluate the assembly quality of reads and validity of markers in *Gynostemma*. In total, 268 primer pairs (74.44%) yielded clear fragments among six *G. pentaphyllum* genotypes. This result matches the 60%–90% success rate reported before. In total, 216 Polymorphic EST-SSR markers were obtained with a polymorphic proportion of 90.38%, which was similar to *Amorphophallus* [26], but was higher than other plants [20,21,52]. Our results suggest that the transcriptome assembly was reliable, and that the EST-SSR markers are usable across 12 species in the genus *Gynostemma*. The observed number of alleles ranged from four to 11 with an average of 6.73, indicating the potential application of these primer pairs. In the present study, EST-SSRs derived from *G. pentaphyllum* and *G. cardiospermum* had a higher transferability rate, which was also observed in other plant taxa [22,25,26,35,64]. It has been proposed that the high transferability rate of EST-SSRs might be due to several factors: (1) the EST-SSRs derived from transcriptome database are conservative when compared with genomic SSRs [65,66]; (2) the more consistent efficiency of amplification of EST-SSRs enhances cross-species transferability [67,68]; and (3) closely-related species benefit from a high SSR transferability rate [26]. However, at the same time, [49] explained that the limitation on the interspecific transfer of SSR markers is caused by homoplasy of band sizes and complex mutational events. The genetic relationship among 26 individuals representing 12 species of *Gynostemma* based on 15 polymorphic SSR loci was clearly shown in dendrogram graph. Two major groups representing *subgen. Gynostemma* and *subgen.* *Triostellum* respectively were identified at a cut-off similarity index of 0.71, the level of genetic similarity was 0.70–1.00, indicating relatively high resolution power and potential utility of polymorphic SSR markers in phylogenetics of *Gynostemma*. As expected, the six *G. pentaphyllum* individuals were classified into three groups, and wild individuals were clustered with cultivated individuals from the same population. The variation between populations was higher than the other *Gynostemma* species, implying that *G. pentaphyllum*, as a widespread species, has a high level of genetic diversity. These results are concordant with previous reports [69]. Therefore, the potential EST-SSRs identified in this study will be an effective tool for germplasm polymorphism assessment or quantitative trait loci mapping in *Gynostemma.*

## 4. Materials and Methods

### 4.1. Plant Materials

Young leaves, flowers and immature seeds from two species in the genus *Gynostemma* (*G. pentaphyllum* and *G. cardiospermum*) were used for RNA extraction and transcriptome sequencing. DNA from 26 individual plants collected from southeast China was used to validate SSR markers and diversity analysis. Detailed information for the plant materials is listed in Table 6.

### 4.2. RNA Extraction, Reverse Transcription and Sequencing

*G. pentaphyllum* and *G. cardiospermum* were collected from two locations of Ankang in Shaanxi province during July 2013 (*G. pentaphyllum*: 32°25′N, 109°04′E; *G. cardiospermum*: 32°13′N, 109°01′E), the multiple individual plants mixture including leaves, stems, flowers, shoot tips and developing seeds for each species were frozen immediately in liquid nitrogen, and stored at −70 °C. After mixing an approximately equal weight of mixture for each species, total RNA was extracted using the TRIzol reagent (Invitrogen, Carlsbad, CA, USA) according to manufacturer instructions, then poly-A mRNA was isolated from total RNA using poly-T oligo-attached magnetic beads (Illumina Inc., San Diego, CA, USA). The quantity and quality of RNA were assessed by gel electrophoresis and spectrophotometry. Purified RNA was used to construct a directional cDNA library using the cDNA Synthesis Kit (Illumina), and then the cDNA library was sequenced using a HiSeq 2000 (Illumina) to obtain short sequences.

### 4.3. Transcript Assembly and Analysis

All raw reads from the two *Gynostemma* species were prescreened to remove adapter sequences, reads with greater than 10% unknown bases, and reads with an average base quality less than 30. High-quality filtered transcriptome reads were assembled into contigs by *de novo* assembly using Trinity tools [34]. A nonredundant set of unigene sequences was then created using paired-end reads by further alignments of the contigs from each species. To annotate them, all unigenes were searched against NCBI’s nonredundant protein (nr) database and Swiss-Prot protein databases using BLASTx with an E-value <10^−5^. The Blast2GO program [70] was used to get Gene Ontology (GO) terms to describe gene products according to three ontologies: molecular function, biological process and cellular component [71]. The unigene sequences were also aligned to the COG database to predict and classify functions. To further understand the biological functions and interactions of genes, pathway assignments were performed based on the Kyoto Encyclopedia of Genes and Genomes (KEGG) database [72] using BLASTx with an E-value threshold of 10^−5^. 

### 4.4. EST-SSR Detection and Pprimer Design

Nonredundant unigene sequences longer than 1000 bp were used for mining SSR loci using the MISA tool [49], and primers were designed using BatchPrimer3 v1.0 software with default parameters [73]. Only cDNA-based SSR loci containing two to six nucleotide motifs were considered, the criteria for selection of SSRs were a minimum of six repeats for di-nucleotide motifs and five repeats for tri-, four repeats for tetra-, penta-, and hexa-nucleotide motifs. Mononucleotide repeats and complex SSR types were ignored. Frequency of SSR refers to the average number of kilobase pairs of cDNA sequence containing one SSR. The parameters for designing PCR primer pairs from sequences flanking SSRs were as follows: (1) primer length range from 18 to 25 bases (optimal 20 bases); (2) PCR product size range of 100 to 300 bp (optimal 200 bp); (3) annealing temperature of 50–60 °C (optimal 55 °C); and (4) a GC content of 40%–60% (optimal 50%). Other parameters were set at the default value of BatchPrimer3v1.0. 

### 4.5. Plant DNA Extraction, PCR Conditions and Separation of SSR Markers

26 individuals, representing 12 *Gynostemma* species (Table 6), were selected for analysis of intraspecific genetic diversity, cross-species amplification with the EST-SSRs, and interspecific relationships. Plant DNA was extracted from leaf samples using the CTAB method [74], and DNA integrity was checked via electrophoresis on 1.5% agarose gel. PCR amplifications were carried out using a MyCycler™ Thermal Cycler (Bio-RAD, CA, USA) in a 10 µL final volume containing 1 × PCR buffer [10 mM Tris-HCl (pH 8.4), 1.5 mM MgCl_2_], 0.2 mM dNTPs, 0.2μM of each primer, 50 ng of genomic DNA, and 0.5 U *Taq* polymerase (Biostar, New Taipei, Taiwan). The PCR reaction program was: DNA denaturation at 95 °C for 5 min; followed by 32 cycles of 95 °C for 40 s, 50–60 °C (depending on optimized annealing temperature) for 30 s and 72 °C for 50 s. The final extension was performed at 72 °C for 10 min. PCR products were analyzed using 8% PAGE and silver stained [75] with a PBR322 DNA marker ladder (Tiangen Biotech, Beijing Co., Ltd., Beijing, China) for assessing the length of the DNA bands. A total of 360 genic SSR markers were selected randomly for genotyping six *G. pentaphyllum* samples from three populations, 30 highly polymorphic loci were selected for testing the transferability of EST-SSRs to the other ten species in the genus *Gynostemma.*

### 4.6. Genetic Analysis and Data Scoring

Of the 30 highly polymorphic loci, genic-SSR markers that amplified successfully in all 12 species were used to assess the genetic diversity in a set of 26 individual plants. Each allele was scored as present (1) or absent (0) for each of the SSR loci. The polymorphism information content (PIC) of each SSR primer was calculated to estimate the allelic variation of SSRs in the 26 individuals according to the formula: $\text{PIC}=1-\Sigma_{i=0}^{\text{n}}{\text{Pi}}^{2}$, where Pi is the frequency of the i^th^ allele for a given SSR marker, and n is the total number of alleles detected for that SSR marker [76]. The genetic similarity between any two individuals was estimated based on Jaccard’s similarity coefficient. All 26 individuals were clustered with the UPGMA algorithm and SAHN procedure of the NTSYS-PC v2.10t [77]. Bootstrapping analysis with 1000 replicates was carried out using the software FREETREE V.0.9.1.50 [78]. Bootstrap values over 50 were considered significant and provided on the dendrogram.

## 5. Conclusions

In this study, we used high-throughput sequencing to characterize the transcriptomes of two *Gynostemma* species. A large-scale EST dataset with 71,607 nonredundant unigenes from *G. pentaphyllum* and *G. cardiospermum* was established, which provided valuable sequences for the discovery of new genes and EST-SSR markers. These results support the view that NGS is a fast and cost-effective approach for gene discovery and molecular marker development in nonmodel species.

## Supplementary Materials

Supplementary materials can be accessed at: http://www.mdpi.com/1420-3049/20/12/19758/s1.
